# Post-January 6 deplatforming shows long-term effects on ideological polarization among Twitter users

**DOI:** 10.1093/pnasnexus/pgaf333

**Published:** 2025-10-22

**Authors:** Cody Buntain, Maria Snegovaya

**Affiliations:** College of Information Studies, University of Maryland, 130 Campus Dr 4th Floor, College Park, MD 20742, USA; Edmund A. Walsh School of Foreign Service, Georgetown University, CC 301, 37th St NW & O St NW, Washington, DC 20057, USA

**Keywords:** deplatforming, polarization, differential effects

## Abstract

This study examines audience response to Twitter’s “Great Deplatforming” following the January 6 attack on the US Capitol. Using a survey of Twitter users from the 2020 US election, we test whether Twitter’s deplatforming coincides with significant changes in respondents’ behavior, operationalized using individuals’ posting frequency and ideological lean—measured daily via a news-sharing metric we validate. To control for exogenous changes in the information environment, we pair Twitter respondents with Reddit accounts that exhibit similar pre-event behavior. Results indicate a shift in Twitter’s ideological landscape: Respondents become more extreme, and polarization between liberals and conservatives intensifies as ideologically extreme users move further from the center. As the Great Deplatforming recedes, our Twitter users trend toward the center, suggesting that, while the platform experiences an immediate shock, it ultimately undergoes a long-term moderating effect. Importantly, these effects do not appear on Reddit, which instead exhibits an immediate reduction in ideological extremity following January 6th and sees no changes in longer-term trends. We also do not find evidence of a uniform flight from Twitter; only conservative-leaning respondents are significantly less likely to remain active. While results show ideologically extreme Twitter accounts experience some suppressive effect in activity, these effects seem specific to the most extreme actors, as decreasing trends among middle-of-the-road actors are present in both Twitter and Reddit. Taken together, while Twitter did experience a significant increase in the level of polarization and ideological extremity, the long-term trend appears to be one of moderation, an effect absent from Reddit.

Significance Statement “Deplatforming” has been a common content-moderation strategy, yet studies on its efficacy have found mixed results. Our study provides new insights by examining individual-level impact of Twitter’s “Great Deplatforming” in January 2021. Previous studies of deplatforming around this event have mainly focused either on aggregated online behavior or on users already active on alternative platforms. Our study instead investigates differential effects among Twitter users from across the ideological spectrum gathered from a survey of political attitudes. This study also contextualizes results on Twitter through a controlled comparison against behavior on a different platform, i.e. Reddit. This analysis is important to the larger context of content moderation and yields better insight into expected effects of deplatforming in a politically contentious context.

## Introduction

Between the elections of 2016 and 2020 and the COVID-19 pandemic, mainstream online social spaces have taken increasingly strident steps to address quality issues on their platforms. These platform interventions have run the gamut from algorithmic suppression/“de-recommendation” of potentially harmful content (1) to blocking influential but extreme content creators (2) to banning whole communities (3). Such content moderation and related “deplatforming” efforts have culminated in the banning of then President Trump from Twitter, Facebook, and other platforms following the January 6th insurrection in the United States in 2021.

While many studies have examined content moderation, the January 6th insurrection and related deplatforming effects are unique in their scale and interactions with the broader US society and political system, and pose specific questions around deplatforming and content moderation in the face of antagonistic political elites. Despite this import, our understanding of the effects and efficacy of deplatform is limited: While we have broad empirical support for prosocial outcomes around moderation (e.g. (1–4)), studies around the 2021 Great Deplatforming have found little positive effect (5) and potentially harmful increased exposure to extreme, alternative spaces (6). This divergence may be partly explained by how studies of the Great Deplatforming have focused either on aggregated online behavior of social media users (e.g. (6)) or on users already active on alternative platforms ((5) or (7)). One potential path to reconcile these contradictory findings is to investigate the differential effects of this deplatforming among specific groups, which has not yet been sufficiently analyzed.

This study offers unique evidence in that regard. By leveraging a survey of Twitter users during the 2020 election period, we are able to trace how the Great Deplatforming affected Twitter engagement patterns among respondents of various self-reported ideological leanings. Specifically, we assess the degree to which polarization, ideological extremity, and posting frequency change following the Great Deplatforming among respondents of various ideological leanings. Critically, we contextualize these Twitter-specific results by comparing these behaviors to a matched set of accounts on Reddit over the same timeframe. To assess polarization and ideological extremity, we use a news-domain-based method for measuring an account’s ideology—validated against self-reported ideological lean in the survey—to track daily changes in ideological sharing.

With respect to Twitter, in the short term, we see significant increases in the level of polarization and ideological extremity immediately following the Great Deplatforming (on par with moving from the Washington Post to HuffPost). In the long term though, ideological extremity on Twitter trends towards the center as the Great Deplatforming recedes into the past. These results are not reflected in Reddit, where we instead only see an immediate level change in polarization, as matched actors on Reddit become more liberal (though these effects are more dependent on the timeframe of observation, while Twitter results are more temporally consistent).

Whether these results are attributable to a disengagement from Twitter, as suggested by Buntain et al. (6), is unclear: Our results show that actors exhibit an immediate increase in posting following the Great Deplatforming, with more ideologically extreme users showing a sharper rise in post frequency. However, by our final round of data collection, fewer conservative accounts remained active on Twitter. Again, Reddit exhibits different behavior, where we see an overall declining trend in posting.

On the whole, while we do see evidence of an immediate increase in polarization and ideological extremity on Twitter following the Great Deplatforming, findings suggest the long-term trend is one of moderation. These findings can help social media platforms understand the differential effects of deplatforming, yielding insights into how it might be employed in conjunction with other interventions to reach unsupported audiences.

## An inconsistent body of related work

In recent years, the loss of commonly held standards of information mediation and the lower threshold of information access has contributed to a rapid spread of partisan-driven, unsubstantiated, false information (8, 9). These channels often achieve surprising levels of success in spreading misleading narratives on Twitter and other social networks (10, 11). For example, in the United States, Donald Trump’s ability to directly reach millions of followers and promote his political agenda played a notable role in his electoral victories (12–14). These actors often mislead and polarize the public and promote extreme tactics, such as violence at political rallies, to achieve their objectives. To enhance user safety, social media platforms have resorted to deplatforming—a practice of suspending or suppressing offensive, extremist and otherwise dangerous users. Assessing the effectiveness of this intervention is crucial for balancing societal safety while preserving the right to free speech (15).

Despite platforms’ increasing reliance on this intervention, academic consensus has yet to be reached on the effectiveness of deplatforming. Some studies have found deplatforming an overall ineffective intervention at improving the information space (16) because deplatformed users might migrate to alternative, less regulated platforms (5, 6) where they are exposed to more-radical content (17, 18), become more polarized (15) and toxic (19). For example, Childs et al. (20) demonstrates that, despite YouTube’s deplatforming antisocial content, extreme YouTube creators maintained a catalog of such content on BitChute (a toxic and alternative video-sharing platform), and these BitChute videos were amplified by regular Twitter users.

In contrast, other studies have found deplatforming interventions to have a significant suppressive effect on dangerous or misleading content (4, 6, 21), as well as decreasing activity and toxicity levels (2, 7). For instance, Reddit’s bans of racist and fat-shaming communities (3, 22); Facebook’s ban of conspiracy theorists (23), and YouTube’s ban of far-right channels (4) have been found fairly effective in limiting the spread of such content. Specific to the Great Deplatforming, McCabe et al. (24) finds that Twitter users who followed deplatformed users tended to share less misinformation after the event.

One path toward reconciling this inconsistency in findings is by examining differential effects *across individuals*. In politically contentious contexts, such as the US January 6th insurrection, partisan leaning potentially mediates the impact of deplatforming across audience segments. Even if deplatforming’s overall effects among all users are significant, the intervention is unlikely to be effective when it fails among individuals particularly prone to engaging with banned content. For example, conspiracy communities might be more resilient to deplatforming policies and more likely to migrate to other platforms as opposed to moderates (15). However, few studies have explored differential impact of deplatforming on individuals of different political leanings. That is because, by design, these studies usually track aggregated behavior of social media users.

Even when accounting for these differential effects, however, disagreement remains, as with a recent study from Meta/its collaborators published in *Science* and the academic response to that study: Guess et al. (25) investigated the impact of algorithmic feed curation in Facebook and Instagram in a 3-month period centered on the 2020 election, finding limited to no impact on polarization, political knowledge, or other key attitudinal measures. Kupferschmidt (26) outlines concerned response to this piece from other academics who raise doubts about the generalizability of the results in Guess et al. (25) given the emergency “break glass” measures Meta was taking at the time of the study. These concerned then drove editors from *Science* to address them in a editorial (27). A key issue in the debate around that article is the small timeframe around which the study assessed impact.

Our study fills these gaps through an analysis of a novel dataset based on a uniquely timed survey of Twitter users run during the 2020 election. Following the 2021 January 6th, insurrection and the attack on the US Capitol, Twitter banned several thousand accounts (the so-called “Great Deplatforming”), ostensibly to limit misinformation about voter fraud and suppress calls for violence. Specifically, on January 8 Twitter suspended the accounts of former President Donald Trump and about 70,000 associated users. The ban has provided a timely opportunity to leverage our survey to investigate partisan groups’ individual-level behaviors following this deplatforming.

## Research questions and hypotheses

Unlike prior work, we focus on heterogeneous effects of the deplatforming based on respondents’ self-reported ideology. In particular, we explore:

- RQ1: To what degree did Twitter users’ posting frequency change following the Great Deplatforming?
- RQ2: To what extent did Twitter users become more or less polarized following the Great Deplatforming?

In an ideal case, this intervention would decrease polarization but not at the cost of user engagement. In other words, users would ideally become less polarized or ideologically extreme without disengaging from the platform. Alternatively, if the Great Platforming drove a more anti-social outcome, as suggested in Buntain et al. (6), we should see a marked increase in polarization or a decline in posting frequency, consistent with individuals either disengaging or leaving to more extreme platforms, as shown in Ribeiro et al. (5) for Parler. We also anticipate a degree of ideological asymmetry in our results, as conservative actors at that time have claimed Twitter to be politically biased against President Trump (part of a long-standing claim that social media platforms are biased against conservatives).

## Methodology

### Modeling changes in polarization and engagement

This study examines the ways in which Twitter users altered their behaviors in the aftermath of the Great Deplatforming, focusing specifically on polarization and engagement on the platform. To investigate these research questions, we use a comparative interrupted time series (ITS) design, similar to Buntain et al. (1). This design models changes in engagement and polarization as functions of the prior day’s posting behavior, the Great-Deplatforming intervention, respondents’ ideological lean, and respondents’ ideological extremity. To assess polarization, we fit two separate models, one for daily ideological lean, and a second for daily ideological extremity (the absolute value of ideological lean, thereby grouping respondents based on the self-reported instensity of lean). We use 2021 January 8 as the date of the Great Deplatforming intervention, as this date is when President Trump was removed from the platform.

The core ITS model is shown in Eq. 1, where the outcome variable $y_{t,a}$ represents polarization/engagement on day *t* for a given account *a*. As is typical for ITS models, we represent these outcomes as a function of time *T* in days since the beginning of our observation and an indicator variable $X_{t}$ denoting whether time *t* falls before or after the intervention. We extend this standard model with an indicator variable representing an immediate, short-term pulse $P_{t}$ in behavior that is coded as 1 only on the day of the intervention. Further, our outcome variables exhibit autocorrelation, so we include $y_{t-1,a}$ to represent this same quantity on the previous day. Beyond these standard factors, we anticipate responses to the Great Deplatforming are heterogeneous across the political spectrum—e.g. conservative Twitter users are more likely to see the intervention as hostile to their party and will therefore respond differently from liberal users. Similarly, we expect that moderate or “middle-of-the-road” users are less likely to react strongly compared to those with more pronounced ideological leanings. As such, we include a factor for ideological lean $I_{a}$ for each account and interact this term with the remaining ITS terms, as shown in Table 1. Of particular interest for this analysis are the treatment and pulse terms, the interaction between treatment and trend (which captures changes in slope attributable to the intervention), and the interactions with ideology—these last factors capture heterogeneous effects.


(1) $$\begin{array}{rl} y_{t,a} & =\beta_{0}+\beta_{1}y_{t-1,a}+\beta_{2}I_{a}\\ & \;+\beta_{3}T+\beta_{4}P_{t}+\beta_{5}X_{t}+\beta_{6}X_{t}T\\ & \;+\beta_{7}I_{a}\cdot T+\beta_{8}I_{a}\cdot P_{t}+\beta_{9}I_{a}\cdot X_{t}+\beta_{10}I_{a}\cdot X_{t}T. \end{array}$$

**Table 1.** Definitions of terms in the ITS model.

	Name	Meaning
$I_{a}$	Ideology	Self-reported ideological lean, from [−3,+3] for strong liberal to strong conservative
*T*	Trend	Days since the beginning of the observation period
$P_{t}$	Pulse	Indicator that is 1 on the day of the intervention, zero elsewhere
$X_{t}$	Treatment	Indicator that is 1 on the day of and all days after the intervention, zero elsewhere

Though this design is relatively standard for ITS models (28), such models can be confounded by history bias. This threat is particularly salient during this timeframe, as many different major events coincided with the Great Deplatforming—see McCabe et al. (24) for a thorough discussion of the difficulty in causal interpretations in this exact context. Hence, to account for factors external to Twitter as a platform, we replicate this model on a comparative set of account behaviors from Reddit, as users on Reddit are similarly exposed to the exogenous factors such as the news cycle, coverage of the January 6 insurrection, and related events. This comparison against behavior on Reddit provides a form of location-based control (29).

### A note on ITS versus alternative designs

Our choice of ITS—over other designs, such as difference-in-difference (DiD)—has two chief motivations: First, the ITS model is well-suited to decomposing effects into immediate and longer-term changes, whereas typical DiD designs focus more on aggregate changes. Second, DiD models require control groups, which are not readily available within Twitter, as the entire platform has some degree of exposure to the effects of the Great Deplatforming as an intervention. While we do include Reddit behavior as a quasi control, behavior across platforms likely violates the parallel-trends assumption in DiD, particularly with respect to frequency of posting. Instead, the ITS model relies on the preintervention period as the control against which postintervention outcomes can be compared.

### Survey data

To investigate our target research questions, we leverage a survey of Twitter users and their political attitudes. Similar to Guess et al. (25), our survey was fielded around the 2020 US election, from 2020 October 22 to November 12, using Lucid Market Research Limited’s online panel. Participants were recruited through banners on internet sites, email, and Panel Portal to ensure the survey’s high ecological validity. Most participants collected points that were later exchanged for rewards. This study was approved by the Johns Hopkins University Institutional Review Board (IRB; no. HIRB00011409). All participants provided informed consent before taking part in the study. The survey included a question asking the respondent to provide their Twitter handles for future analysis. We defined “Twitter users” as respondents with a Twitter ID who were actively posting on Twitter at least weekly. We then dropped respondents who provided likely fake Twitter handles, such as those belonging to celebrities/organizations, those with over 10,000 followers, or those located outside of the United States, based on their geolocation.

Because of a high and nonrandom attrition (respondents without Twitter handles, those not sufficiently active on Twitter, and those who provided several identical Twitter handle names were dropped from the analysis), this sample does not represent any well-defined or representative US population. However, the survey was designed to be broadly representative of the general US population through age, gender, race, state of residence, and income quotas. We also asked our respondents questions regarding their demographic information, attitudes, and ideological extremity. The survey includes questions about ideological lean, specifically whether respondents identify more closely with liberals or conservatives. After filtering, the final sample consists of 2,061 respondents.

### Respondents’ ideological lean

From this survey data, we constructed two measures of our respondents’ ideology. Self-reported ideology was identified based on the survey question, which asked: “Where would you place yourself on a scale from 1 to 7 where 1 means very liberal and 7 means very conservative?” Responses included: “Very liberal,” “Liberal,” “Somewhat liberal,” “Middle of the road,” “Somewhat conservative,” “Conservative,” “Very conservative,” and “Don’t Know.” We then centered this spectrum around zero, such that it runs from “−3” to “+3” for “Very Liberal” to “Very Conservative.” Table 2 shows the frequencies of these bins, suggesting a slightly liberal average ideological lean among respondents, consistent with past studies of ideology in Twitter (30)—though we note the X incarnation of Twitter has shed a significant percentage of its liberal-leaning audience (31).

**Table 2.** Counts of respondents by ideological lean.

Self-reported lean	Respondents
Very liberal	378
Liberal	298
Somewhat liberal	244
Middle of the road	543
Somewhat conservative	161
Conservative	203
Very conservative	192
Don’t know	59

Results suggest the sample leans liberal, consistent with prior studies of ideological distribution in Twitter.

Separate from ideology, we also measure *ideological extremity* to capture how far from center/moderate respondents position themselves. We measure ideological extremity via the absolute value of one’s self-reported ideological lean (0 to +3). We introduce this measure to capture similar behavior among strongly politically engaged actors, even when they hold fundamentally opposing ideologies.

### Collecting Twitter data

Using Twitter’s now-defunct Researcher API and list of accounts provided by our survey respondents, we have collected their Twitter posts over a 2-year period, covering January 2020 to January 2022. This collection thus covers user behavior in the year prior and the year following the January 6th insurrection and ensuing Great Deplatforming. This collection occurred across multiple waves, where we ran our first collection immediately following survey completion in early December 2020, covering 2020 January 1 to December 1. We updated this collection three times, once in January 2021, again in March 2021, and finally in April 2022, resulting in a final set of 2,872,124 tweets from 1,778 Twitter accounts.

While these dates provide a large pre- and postintervention observation period, the gap in collection between March 2021 and April 2022 introduces limitations around when individual accounts left the platform. Consequently, of the original 1,778 accounts, 1,335 remained active on the platform in April 2022—“active” here means having posted content between 2021 January 1 and 2022 January 6 and the account was not deleted or suspended in April 2022. The remaining 443 accounts may have left Twitter, deleted their account, or simply not posted in this timeframe. As intimated in McCabe et al. (24), these inactive accounts were more likely to belong to respondents who self-identify as conservative-leaning.

### Imputing daily ideological lean via news sharing

To measure changes in daily ideological lean, we employ a news domain-based method for assessing a user’s account ideological characteristics. Using a domain-level ideology scaling presented in Robertson et al. (32), which strongly parallels other ideology measures (6), we infer a daily “ideological lean” score for each respondent’s account. As in the well-established NOMINATE method Poole and Rosenthal (33), negative scores correspond to liberal ideological positions, and positive scores correspond to conservative positions. This account-level score is the weighted average of domain ideology scores shared on that day, following Bond and Messing (34), who propose a similar method for assessing individual-level ideological lean via social media behavior. That method first imputes ideological scores for US politicians on the Facebook platform based on co-follower audiences, and then propagates these values to individuals using the weighted average of the politician pages they follow. In our work, we use a similar method wherein we leverage extant domain-level ideology scores from Robertson et al. (32) in place of the politician scores from Bond and Messing and use the same weighted averaging approach to impute account-level measures for ideological lean. This metric allows us to measure changes to ideological lean over time at a daily level. For modeling purposes, if an account does not share political news on a particular day, we include a placeholder value denoting no estimate for that day. We also do not impose a minimum threshold on content type or the necessary number of shares per day, as such thresholds would further bias our data; most respondents post relatively few times per day with the exception of very-liberal and very-conservative accounts (see Fig. 2). That said, we have re-run this analysis at the weekly level and produced similar results; code for replicating these analyses are available in the Supplementary material.

To further validate our metric, we compare it with respondents’ self-reported ideological leans for those who selected an answer other than “Don’t Know” and whose accounts shared hyperlinks to news in 2020, resulting in a sample of 1,094 respondents. For this comparison, we calculate each account’s aggregate ideological lean by averaging across each day in 2020. This calculation differs from the aggregate metric in Robertson et al. (32), where each share of a domain contributes equally to the aggregate value, to make our metric robust toward large bursts in activity; i.e. even if an account shares more political news on 1 day than another, both days contribute equally to that account’s aggregate score. We then take the average of this daily aggregated mean across all accounts with a given self-reported lean and compare it against self-reported lean. As shown in Table 3, our average score for ideological lean aligns perfectly with the respondent’s self-reported lean. This consistency forms the basis for our investigation into how deplatforming impacts one’s observed ideological lean.

**Table 3.** Self-reported versus domain-based ideological lean.

Self-reported lean	*n*	Mean domain-based lean
Very liberal	222	− 0.043
Liberal	170	− 0.015
Somewhat liberal	127	0.006
Middle of the road	303	0.059
Somewhat conservative	76	0.068
Conservative	99	0.080
Very conservative	97	0.120

These measures cover 1,094 respondents who shared hyperlinks to news in 2020. Rankings of self-reported and domain-imputed scores align completely.

**Fig. 1.**
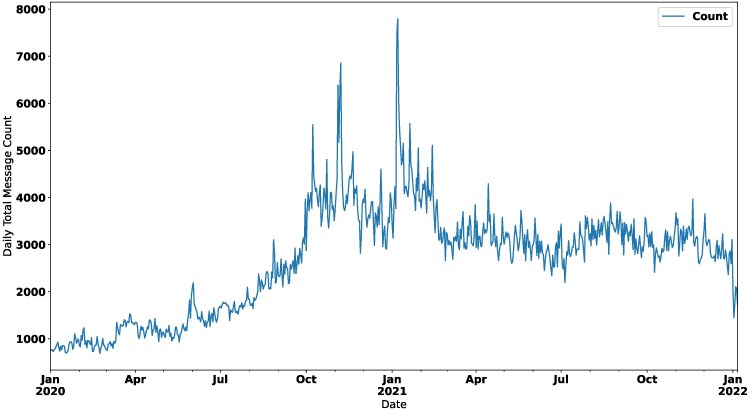
Daily tweet count from 793 accounts. Activity peaks on 2020 November 4–8 and again on 2021 January 6–7.

**Fig. 2.**
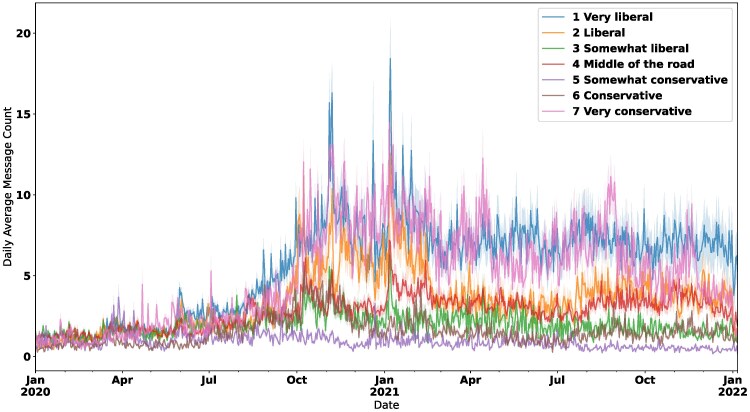
Daily average post count by ideological lean. Hyper-partisan accounts (i.e. respondents who self-identify as “Very Liberal” and “Very Conservative”) are generally the most active.

To scale this measure to all our data from 2020 January 1 to 2021 December 31, we further restrict the set of respondents for whom we can impute daily ideological lean: Of the 1,335 accounts that remained active during our final collection in April 2022, only 952 shared hyperlinks to news in 2021, and 793 respondents shared hyperlinks to news in both regimes. This final set includes 1,967,990 tweets with daily post frequencies peaking in late 2020/early 2021, as shown in Fig. 1. Figure 2 shows the daily activity across these bins, wherein extreme/hyper-partisan accounts—“strong liberal” and “strong conservative”—are the most active, posting about twice as often as respondents in other ideological bins, with differences across bins widening after the election.

**Fig. 3.**
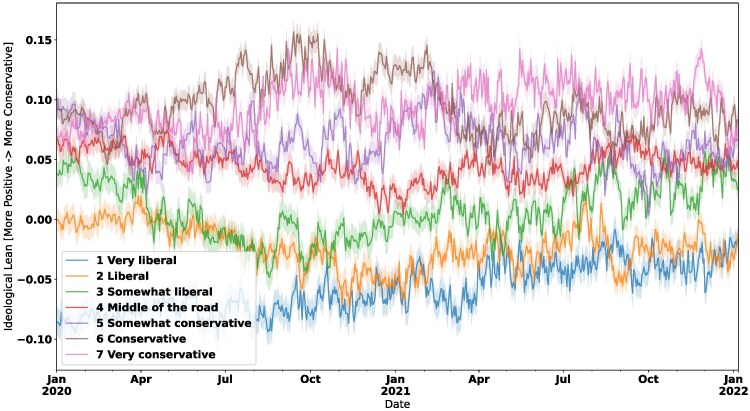
Daily average imputed ideological lean by self-reported bin.

Figure 3 then shows how these respondents’ ideological lean changes over time, grouped by self-reported lean. Middle-of-the-road respondents tend to stay the most centrist, whereas more ideologically extreme groups (very liberal/liberal and very conservative/conservative) tend to stay around the same space. The “somewhat liberal/conservative” groups start and end near the middle-of-the-road groups but occasionally move toward more partisan positions before coming back.

### Matching Twitter respondents to reddit accounts

**Fig. 4.**
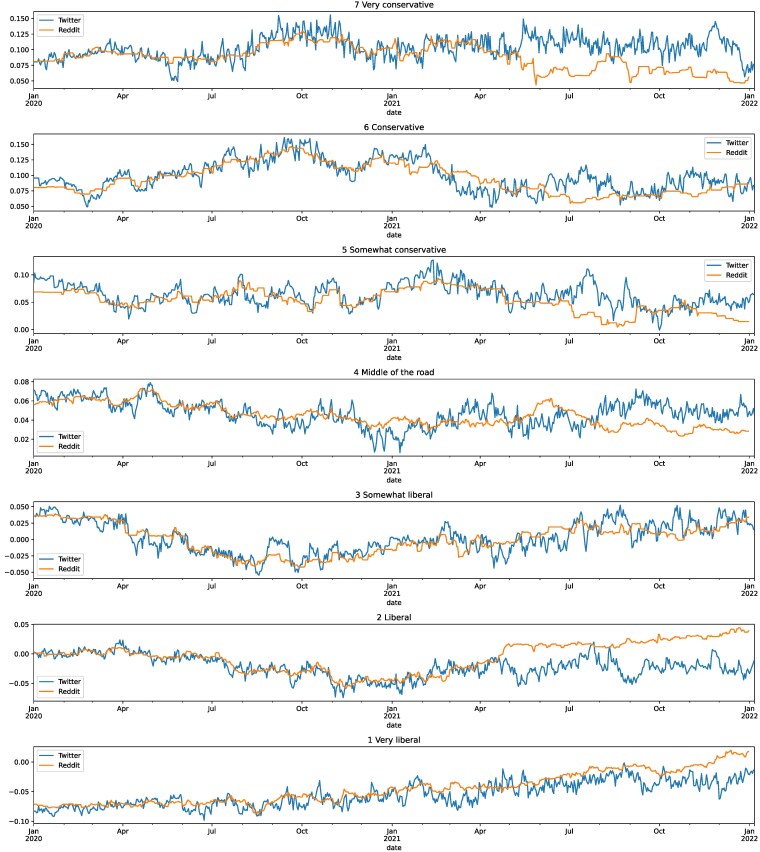
Daily average versus self-reported lean by per platform. The blue lines represent metrics from our Twitter respondents, and the orange lines represent matched Reddit accounts; poster January 2021, curves appear to diverge between the two platforms.

To assess whether outcomes are specific to Twitter or reflect broader shifts in the news ecosystem and post-election disinterest, we match survey respondents’ Twitter accounts to accounts extracted from Reddit using the Reddit Pushshift dataset (35). Using contemporaneous data from January 2020 to December 2021, we have extracted daily measures of ideological lean from 763,302 Reddit accounts. We restrict this data to only behavior from 2020 and, for each account in our Twitter dataset, we find the corresponding Reddit account with the smallest Euclidean distance between vectors of daily ideological lean. This matching process produces 762 matches between Twitter respondents and Reddit accounts, with an average distance of 1.4354 between them. Figure 4 shows the aligned daily ideological trends from these matched accounts, by self-reported ideological lean.

## Results

### Posting frequency

Our first research question explores the degree to which users on Twitter increased or decreased their activity during and after the Great Deplatforming. To examine this intervention, we look to the results from fitting the linear regression model from Eq. 1, where we model daily post frequency as a function of ideological lean, ideological extremity (i.e. the absolute value of this lean), and both factors combined. Table 4a and b show results for these models fit with data from 762 survey respondents on Twitter and Reddit respectively.

**Table 4.** Changes in log-transformed daily post frequency by ideological lean and extremity

	(a) Twitter					
	Model 1		Model 2		Model 3	
	B	SE	B	SE	B	SE
Trend (*T*)	0.7560	0.062***	0.2264	0.080**	0.2321	0.080**
Pulse (*P*)	− 0.0844	0.465	0.1280	0.872	0.1038	0.873
Treatment ($X_{t}$)	0.6963	0.106***	0.1761	0.136	0.1771	0.136
Slope ($X_{t}T$)	− 0.8920	0.092***	− 0.2566	0.120*	− 0.2599	0.120*
Ideology (*I*)	0.0150	0.013			0.0015	0.014
Ideology·Trend (IT)	− 0.1302	0.039**			− 0.0718	0.040
Ideology·Pulse (IP)	0.3408	0.240			0.3192	0.241
Ideology·Tx ($IX_{t}$)	− 0.0672	0.068			− 0.0092	0.070
Ideology·Slope ($IX_{t}T$)	0.1093	0.059			0.0387	0.060
Extremity (|I|)			− 0.0825	0.019***	− 0.0820	0.020***
Extremity·Trend (|I|T)			0.3865	0.055***	0.3566	0.055***
Extremity·Pulse (|I|P)			− 0.2611	0.449	− 0.1261	0.455
Extremity·Tx ($|I|X_{t}$)			0.3570	0.098***	0.3536	0.100***
Extremity·Slope ($|I|X_{t}T$)			− 0.4462	0.084***	− 0.4303	0.086***
Adj $R^{2}$	0.730		0.730		0.730	
AIC	3,843,004		3,842,859		3842814	
${\;}^{*}$ P<0.05, ${\;}^{**}$ P<0.01, *** P<0.001						

	(b) Reddit					
	Model 1		Model 2		Model 3	
	B	SE	B	SE	B	SE
Trend (*T*)	0.0131	0.006*	− 0.0003	0.010***	0.0002	0.010
Pulse (*P*)	− 0.0731	0.032*	− 0.0503	0.046	− 0.0518	0.045
Treatment ($X_{t}$)	0.0490	0.009***	0.0525	0.015***	0.0523	0.015***
Slope ($X_{t}T$)	− 0.0477	0.008***	− 0.0451	0.013**	− 0.0452	0.013**
Ideology (*I*)	0.0018	0.002			3.2E-6	0.002
Ideology·Trend (IT)	− 0.0073	0.003*			− 0.0059	0.003
Ideology·Pulse (IP)	0.0208	0.026			0.0185	0.022
Ideology·Tx ($IX_{t}$)	0.0032	0.005			0.0028	0.005
Ideology·Slope ($IX_{t}T$)	0.0014	0.005			0.0011	0.004
Extremity (|I|)			− 0.0112	0.003***	− 0.0112	0.003***
Extremity·Trend (|I|T)			0.0112	0.006*	0.0088	0.005
Extremity·Pulse (|I|P)			− 0.0222	0.041	− 0.0144	0.034
Extremity·Tx ($|I|X_{t}$)			− 0.0034	0.009	− 0.0022	0.008
Extremity·Slope ($|I|X_{t}T$)			− 0.0021	0.008	− 0.0017	0.007
	0.470		0.470		0.470	
	1,323,717		1,323,720		1,323,693	

${\;}^{*}$ P<0.05, ${\;}^{**}$  P<0.01, *** P<0.001

Focusing on both platforms’ model 3, which has the lowest Akaike Information Criterion (AIC), these ITS results demonstrate differences in effect across partisan groups and across platforms. First, we note that, regardless of intervention, Twitter shows a significant, positive effect on the trend in activity, consistent with our observation that Twitter actors are becoming more active over the 2-year period. Similarly, regardless of intervention, ideologically extreme actors began with, on average, lower activity relative to their middle-of-the-road counterparts, as seen with a significant negative effect on ideological extremity—an effect mirrored in Reddit. Twitter also sees a positive and significant effect on the interaction between extremity and trend, suggesting that, while extreme actors were originally less active than moderates, these partisans became increasingly active over time in the lead up to January 2021. Focusing on the timeframe following the Great Deplatforming, we see a common negative effect on the post-intervention slope ($X_{t}T$) on both Twitter and Reddit, suggesting actors became less active over time, regardless of ideological lean. As this effect appears in both platforms, it likely should not be attributed to Twitter’s intervention.

Specific to Twitter and the impact of the Great Deplatforming, we find a positive and significant effect on the interaction between extremity and treatment, showing a positive shift in the level of activity from partisan actors; that is, increasingly extreme actors immediately became more active on Twitter following 2021 January 8. Offsetting this increase, we also see a significant suppressive effect on the interaction among extremity and post-intervention slope, suggesting that extreme actors posted less frequently as the Great Deplatforming receded into the past relative to moderates. These results are inconsistent with hypotheses suggesting that the Great Platforming suppressed activity on Twitter, as suppressive effects appear disconnected from the underlying platform, and only extreme Twitter uses fell in posting activity.

As a post hoc analysis, we also count occurrences of the alternative platforms outlined in Buntain et al. (6)—Gab, BitChute, and Parler—and find little to no sharing of links to these spaces. Only 16 accounts in our sample have shared links to these alternative platforms in the predeplatforming period; that number increases to 23 accounts post-deplatforming, but only two share these links more than 10 times. Hence, our results show little evidence of disengagement or platform migration—though exposure to these alternative platforms might still remain an issue, as Buntain et al. (6) suggest.

### Sharing by ideological lean and extremity

Turning to trends in sharing by ideological lean and extremity shown in Fig. 4, Table 5 shows deplatforming’s impact on Twitter (Table 5a) with a comparison against our matched Reddit accounts (Table 5b). In general, Twitter shows a respondent’s self-reported ideological lean and extremity are significant and positive predictors of their daily behavior on Twitter, providing a reasonable face validity check for our daily metrics—though this consistency does not hold among our matched Reddit users. For daily ideological lean, we see a positive trend (I⋅T), suggesting that, over the observation period, the platform becomes more ideologically polarized—that is, liberals and conservatives become increasingly ideologically divided. Daily extremity similarly shows an overall increasing trend; i.e. Twitter was already increasingly extreme day-by-day during our observation period. These patterns do not emerge among our Reddit-matched accounts.

**Table 5.** Models of daily ideological lean and ideological extremity based on self-reported ideological lean.

	(a) Twitter				(b) Reddit			
	Daily ideology		Daily ideological extremity		Daily ideology		Daily ideological extremity	
	B	SE	B	SE	B	SE	B	SE
Trend (*T*)	− 0.0026	0.005	0.0175	0.005***	0.0012	0.007	0.0045	0.005
Pulse (*P*)	− 0.0300	0.019	− 0.0094	0.02	− 0.0124	0.027	− 0.0510	0.031
Treatment ($X_{t}$)	− 0.0083	0.007	0.0311	0.007***	0.0254	0.015	− 0.0311	0.015*
Slope ($X_{t}T$)	0.0064	0.006	− 0.0302	0.006***	− 0.0256	0.013	0.0199	0.011
Ideology (*I*)	0.0115	0.002***			0.0019	0.002		
Ideology·Trend (IT)	0.0059	0.003*			0.0008	0.005		
Ideology·Pulse (IP)	− 0.0039	0.013			0.0259	0.016		
Ideology·Tx ($IX_{t}$)	0.0091	0.004*			0.0097	0.011		
Ideology·Slope ($IX_{t}T$)	− 0.0068	0.004			− 0.0105	0.009		
Extremity (|I|)			0.0071	0.002***			0.0014	0.002
Extremity·Trend (|I|T)			0.0014	0.003			− 0.0060	0.004
Extremity·Pulse (|I|P)			0.0009	0.013			0.0486	0.027
Extremity·Tx ($|I|X_{t}$)			− 0.0006	0.004			0.0082	0.010
Extremity·Slope ($|I|X_{t}T$)			0.0024	0.004			− 0.0021	0.008
Adj $R^{2}$	0.320		0.217		0.954		0.949	
AIC	− 26,593		− 49,513		− 6,191		− 7,820	
	${\;}^{*}$ P<0.05, ${\;}^{**}$ P<0.01, *** P<0.001				${\;}^{*}$ P<0.05, ${\;}^{**}$ P<0.01, *** P<0.001			

Looking to the impact of the Great Deplatforming specifically, we see a significant positive effect on the level of ideological polarization ($I\cdot X_{t}$), showing that, following Twitter’s intervention, the level of polarization on Twitter increased immediately, and these effects compound as one moves away from the ideological center; that is, respondents with stronger ideological leans move farther from the center. Effects on daily extremity show a more mixed outcome, however: We observe an immediate increase in the level of extremity on Twitter following the Great Deplatforming ($X_{t}$), in contrast to a significant and immediate decrease in extremity among our matched Reddit accounts. At the same time though, we *also* observe a significant moderating effect in the slope of extremity after this intervention ($X_{t}T$), suggesting the whole platform becomes less extreme over time. This effect is absent on Reddit. The relative strength of coefficients on Twitter also suggest that, within a few weeks, the platform returned to preintervention levels of ideological extremity and continued to exhibit a moderating trend.

## Discussion and conclusions

In this study, we combined an online survey and computational social science techniques to analyze the differential effects of the “Great Deplatforming” on partisan groups on Twitter, using matched Reddit accounts as a comparison group. Our study makes several contributions: First, our news domain-based method for measuring ideology over time provides a valuable approach for estimating temporal changes in ideological polarization. Given the proliferation of domain-level measures (as in Ref. (6) and in data offerings from companies like NewsGuard Technologies^a^), validating this method against self-reported data gives credence to research designs based on similar digital-trace data.

In the broader context of deplatforming, prior studies (5, 6) have raised concerns about whether deplatforming provides sufficient prosocial value to offset potential exposure to more extreme platforms. Our study speaks directly to this concern: While Mekacher et al. (7) find that far-right actors who are on both Twitter and Gettr “are more toxic on Twitter” than they are on Gettr following January 6th, we find that Twitter’s intervention co-occurs with a prosocial, long-term moderating effect, where the entire platform becomes less partisan over time, potentially countering Twitter’s pre-existing trend toward ideological extremity observed in Table 5a. These findings are broadly consistent with other studies of deplatforming, which show evidence of moderation among users who remained on platforms following such interventions (3, 4, 22, 23).

We also find little evidence of additional exposure to extreme platforms, countering the suggestion in Buntain et al. (6). Our post hoc analysis shows very limited additional engagement with platforms like Gab, BitChute, etc. In contrast, Model 3 from Table 4 suggest more-conservative actors were already becoming less active on the platform prior to the Great Deplatforming, and this trend in activity decreases following the intervention. Crucially, more-extreme accounts, that were increasing in activity prior to the Great Deplatforming, have become less active in the postintervention period. These results are more indicative of self-censorship or overall disengagement with Twitter among extreme accounts, as opposed to platform migration.

We note that the temporal effects observed on Twitter may be attributable to event dynamics, such as those described in Rasmussen and Petersen (36). Our inclusion of matched accounts from Reddit, where no similar platform-scale intervention occurred, allows us to localize certain effects to Twitter versus those that may be driven by political contention in 2020 and 2021. As such, while these results should not be interpreted as explicitly causal, we observe on Twitter a sample-wide postintervention trend away from extremity and toward moderation (in Table 5a). Importantly, our Twitter-specific findings on posting activity are not consistent with overall Twitter users’ disengagement from the platform; instead, we see a main trend of increasing activity from our respondents, and the level of extreme-actor activity increases in the aftermath of this intervention. Reddit, in contrast, is more suggestive of conservative disengagement following the Great Deplatforming.

### Threats to validity

Using observational data like domain sharing for inferring attributes like ideological lean is fraught with potential confounders. In the context of this work, two such issues arise: First, we must consider whether a person’s choice to share a particular news article is indicative of their ideological preferences for that content. As shown in Kim et al. (37), criticism is a major motivation for sharing political content online, so if our survey respondents frequently share links to news articles only to criticize them—i.e. they frequently engage in counter-attitudinal sharing—then estimates of their ideological positions would be incorrect. This possibility is of limited threat in our study, however, as counter-attitudinal sharing is relatively rare compared to party-aligned sharing (38, 39), and ideology estimates based on shares, likes, or views (at least in Facebook) show no significant difference (40). In our analysis, counter-attitudinal sharing would manifest as noise in our estimates, and if one were to assume this phenomenon was consistent across the pre- and post-Deplatforming timeframes, estimates on the impact of Twitter’s intervention should be resilient to this noise. Alternatively, if counter-attitudinal sharing were to increase following the Deplatforming, then Wojcieszak et al. (38) suggests this phenomenon would be more pronounced among ideologically extreme accounts, making them appear more moderate than they actually are. In fact, we observe the opposite: extreme actors become more ideologically extreme following the Deplatforming.

Beyond counter-attitudinal sharing, a second potential threat arises from our collection procedures, as we gather behavioral data at three distinct moments rather than in real time. Consequently, respondents may have shared extreme content in the immediate aftermath of Twitter’s Deplatforming but then deleted that content prior to our collection; again, such behavior would bias our estimates of ideology. This “memory hole” (41, 42) problem is well-documented. If accounts were deleting their more extreme content following Twitter’s Deplatforming, this behavior would manifest as a moderating effect, making accounts appear more moderate in our collections than they actually were in the days immediately following the January 6 insurrection. Table 5 instead shows that Twitter accounts tended to become more polarized in this timeframe, and if we were underestimating ideological extremity because of deletion, then this effect would only be magnified. This memory-hole confounder, however, could explain why we do not observe similar effects on Reddit if Reddit users quickly deleted their content. To investigate this possibility, we examine the delay in the Pushshift Reddit (35) and find that much of the content from January 2021 was collected in June 2021, suggesting a possible memory-hole problem. Unique to Reddit, however, when an author deletes a post, the post does not vanish but is instead replaced with a special “[deleted]” token; using this token to track deletions around the Great Deplatforming, we find a steady month-to-month decline in deletions (23.32% in November, 22.78% in December, 22.24% in January, 21.29% in February, and 20.83% in March). While this deletion percentage is nontrivial, this trend would not explain the moderating effects we observe in Reddit, as one would expect more extreme content to be deleted, and higher deletions in the preintervention period would result in accounts appearing more moderate the further one goes into the past (our models also control for such global trends).

### In closing

While results are mixed, we have evidence that Twitter users did not wholly shift to a more polarized and extreme group for the entire observation period. Instead, although we observe reactive changes in the level of polarization and extremity following the intervention, our results also show that these accounts trended toward moderation as the Great Deplatforming faded into the past. Whether Twitter’s Great Deplatforming was the *best* intervention for content moderation remains a difficult question, but establishing a platform-wide norm stating that election denial and calls for violence are unwelcome on Twitter appears to have effects in the long term at the cost of short-term reaction. Establishing this norm then has potentially useful downstream effects such as limiting markets for extreme content, similar to YouTube’s shift to remove harmful content from recommendation (1, 43).

## Supplementary Material

pgaf333_Supplementary_Data

## Data Availability

Anonymized engagement and ideology data, aggregated at the daily level, along with associated code for inference and modeling are available online at https://doi.org/10.17605/OSF.IO/KQFM6.

## Note

a
http://www.newsguardtech.com

## Preprints

This manuscript was posted on a preprint: https://papers.ssrn.com/sol3/papers.cfm?abstract_id=4739537.
