# Supplementary material for: Post-January 6 deplatforming shows long-term effects on ideological polarization among Twitter users
Source: PNAS Nexus. 2025 Oct 22;4(11):pgaf333. doi: 10.1093/pnasnexus/pgaf333 (PMC12624514; doi:10.1093/pnasnexus/pgaf333)
Supplement: pgaf333_Supplementary_Data [file pgaf333_supplementary_data.pdf]

Welcome! Social media use has skyrocketed in recent years, and they play an increasingly important role in our lives. We are U.S.-based researchers, and we are asking you to help us understand people's media habits and opinions on a number of social and political issues by participating in our study.

If you choose to participate, as part of this study we ask you to provide us your Twitter handle. It is important that your Twitter account is set to public and not to private. (If you haven't changed anything in the settings, it is automatically set to public.)

The survey will take about 10-15 minutes to complete. Your participation in the survey is completely voluntary and all of your responses will be kept confidential.

Thank you for your time and cooperation!

1) **Do you have a Twitter account? [If no - terminated]**

- **Yes**
- **No**

1a) Is your Twitter account set to public or private mode? (If you haven't changed anything in the settings, it is automatically set to public.)
- My account is set to private mode
- My account is set to public mode
- Don't know

2) **Would you be willing to share you Twitter account with us for the purposes of social science research? If so, please provide your twitter handle __________ Twitter handles appear after the @ sign in your profile URL and is unique to your account. [If no - terminated]**

3) **How often are you on Twitter? [If less than once a week - terminated.]**
- Several times a day
- Once a day
- Several times a week
- Once a week
- Once every two to three weeks
- Once a month
- Less often

4) **People use Twitter for different purposes. Here are some topics people often discuss on Twitter. Check all that applies to you:**
- Political issues
- Social issues
- Entertainment
- Sports
- Communicating with friends
- _______ [or fill in the blank]

5) **Gender**
- Male
- Female
- _______ [or fill in the blank]

6) **Are you of Hispanic, Latino, or Spanish origin?**
No, not of Hispanic, Latino, or Spanish origin
Yes, Mexican, Mexican American, Chicano
Yes, Cuban

Yes, Puerto Rican
Yes, another Hispanic, Latino, or Spanish origin *** Argentina
Yes, another Hispanic, Latino, or Spanish origin *** Colombia
Yes, another Hispanic, Latino, or Spanish origin *** Ecuador
Yes, another Hispanic, Latino, or Spanish origin *** El Salvadore
Yes, another Hispanic, Latino, or Spanish origin *** Guatemala
Yes, another Hispanic, Latino, or Spanish origin *** Nicaragua
Yes, another Hispanic, Latino, or Spanish origin *** Panama
Yes, another Hispanic, Latino, or Spanish origin *** Peru
Yes, another Hispanic, Latino, or Spanish origin *** Spain
Yes, another Hispanic, Latino, or Spanish origin *** Venezuela
Yes, another Hispanic, Latino, or Spanish origin *** Other Country
Prefer not to answer

7) What is your race?
White
Black, or African American
American Indian or Alaska Native
Asian *** Asian Indian
Asian *** Chinese
Asian *** Filipino
Asian *** Japanese
Asian *** Korean
Asian *** Vietnamese
Asian *** Other
Pacific Islander *** Native Hawaiian
Pacific Islander *** Guamanian
Pacific Islander *** Samoan
Pacific Islander *** Other Pacific Islander
Some other race
Prefer not to answer

8) What is your year of birth? ____

9) Which language or languages do you speak at home/with your family? [Allow two options]
- English
- Spanish
- Chinese
- Tagalog
- Vietnamese
- French or French Creole
- _______ [or fill in the blank]

10) Which U.S. State do you currently reside in? ______ [multiple choice]

11) What type of community do you live in?
- a large central city (over 250,000)
- a suburb of a large central city
- a medium size city (50,000 to 249,999)
- a suburb of a medium size city
- small city or town (10,000 to 49,999)
- a town or village (2,500 to 9,999)
- rural area less than 10 miles from the closest town
- rural area more than 10 miles from the closest town

12) What is the highest degree or level of school you have completed?

- Less than a high school diploma
- Regular high school diploma
- Vocational school/ union certificate
- Some college, no degree
- Bachelor’s degree
- Master’s degree
- Degree higher than a master’s

13) Information about income is very important to understand how people are doing financially these days. Your answers are confidential. Would you please give your best guess? What is your total household income before taxes?
- Less than $35,000
- $35,000-$24,999
- $25,000-$49,999
- $50,000-$74,999
- $75,000-$99,999
- $100,000-$149,999
- $150,000 or more

14) If you were asked to use one of four names for your social class, which would you say you belong in: the lower class, the working class, the middle class, or the upper class?
- Lower class
- Working class
- Middle class
- Upper class
- Don't know

15) In the last 12 months, has your personal economic situation become _____?
- much better
- somewhat better
- about the same
- somewhat worse
- or much worse

16) Everyone is facing some kind of financial challenge these days. And some are in a better position to handle it than others. So far as you and your family are concerned, would you say that you are pretty well satisfied with your present financial situation, more or less satisfied, or not satisfied at all?
- pretty well satisfied
- more or less satisfied
- rather dissatisfied
- not satisfied at all

17) Compared to your parents when they were the age you are now, do you think your own standard of living is ___ than theirs was?
- much better
- somewhat better
- about the same
- somewhat worse
- or much worse

18) We would now like to ask you some questions about your personal use of news.

Typically, how often do you use the following platforms for information on political and social developments? [Randomize the items]

		Several times a day	Once a day	Several times a week	Once a week	Less often
1.	Television					
2.	News websites or applications					
3.	Printed newspapers					
4.	Radio					
5.	Social media (Twitter, Facebook groups, Reddit, Instagram, Parler, WhatsApp groups, etc.)					
6.	Word of mouth (family / friends / colleagues) – in person/by phone/email					

19) In general, how much do you agree with the following statements? [1-4 randomize the order]

		completely	somewhat	not very much	not at all
1.	The news media pay enough attention to important political topics				
2.	Over time most news media reporting is pretty accurate				
3.	In presenting the news dealing with political and social issues, do you think that news organizations deal fairly with all sides				
4.	The mainstream media is more interested in making money than telling the truth				

20) Based on what you know, how often do you believe the nation's major news organizations fabricate news stories?

- Never
- Once in a while
- About half the time
- Most of the time
- All the time

21) Please, answer the following question. If you're running a race and you pass the person in second place, what place are you in? ___ [2]

22) Please, answer the following question. If A farmer had 15 sheep and all but 8 died. How many are left? ___ [8]

23) Please, answer the following question. If Amy's father has three daughters. The first two are named April and May. What is the third daughter's name? ___ [Amy]

24) Please, answer the following question. If You have a pound of feathers and a pound of iron. Which one weighs more? ___ [The same]

25) Now we are going to ask you some questions about your general attitudes and opinions.

Several groups of people are listed below. For each group, please indicate whether you trust people from this group completely, somewhat, not very much or not at all:

		completely	somewhat	not very much	not at all
	Your family				
	Your neighborhood				
1.	People you know personally				
2.	People you meet for the first time				
3.	People of another religion				
4.	People of another ethnicity				

5.	Jewish people				
6.	Women				
7.	Non-white people				
8.	White people				
9.	LGBTQI people				
10.	Doctors				
11.	Scientists				
12.	Evangelical Christians				
13.	Atheists				

26) For each of the following, please indicate, how much confidence you have in it:

		**completely**	**somewhat**	**not very much**	**not at all**
1.	Major news organizations				
2.	Judicial institutions like Supreme Court				
3.	Ivy League universities				
4.	Global corporations				
5.	International organizations like the United Nations (the UN), the World Trade Organization (the WTO), World Bank				
6.	World Health Organization (WHO)				
7.	The two main political parties				
8.	Elected officials in Washington				
9.	U.S. police				
10.	U.S. military				

27) To what extent do you agree with the following statements? [RANDOMIZATION within each group]

		Strongly agree	Somewhat agree	Neither agree nor disagree	Somewhat disagree	Strongly disagree
1.	Elected politicians lose touch with the people pretty quickly					
2.	The differences between ordinary people and the ruling elite are much greater than the differences between ordinary people					
3.	People like me have no influence on what the government does.					
4.	Politicians talk too much and take too little action.					
5.	The American economy is rigged to advantage the rich and powerful					
6.	The people should have the final say on the most important political issues by voting on them directly in referendums.					
7.	The people should be asked whenever important decisions are taken.					
8.	The people, not the politicians, should make our most important policy decisions.					
9.	The politicians in Congress need to follow the will of the people.					
10.	Politicians should lead rather than follow the people.					
11.	To fix America, we need a strong leader willing to break the rules.					
12.	Ordinary people all pull together.					

13.	Ordinary people are of good and honest character.					
14.	Ordinary people share the same values and interests.					
15.	Although Americans are very different from each other, when it comes down to it they all think the same.					

28) Generally speaking, do you usually consider yourself a Republican, a Democrat, an Independent, or what?
- Strong Republican
- Not very strong Republican
- Closer to the Republican party
- Independent
- Closer to the Democratic party
- Not very strong Democrat
- Strong Democrat
- Other party
- No party
- Not sure

29) Where would you place yourself on a scale from 1 to 7 where 1 means very liberal and 7 means very conservative?
- 1 Very liberal
- 2 Liberal
- 3 Somewhat liberal
- 4 Middle of the road
- 5 Somewhat conservative
- 6 Conservative
- 7 Very conservative
- Don't know

30) What do you feel when you think about Democrats?

		**1 – not at all**	**2**	**3**	**4**	**5**	**6**	**7- very strongly**
**1.**	Angry							
**2.**	Afraid							
**3.**	Hopeful							
**4.**	Enthusiastic							
**5.**	Not relevant to me							

31) What do you feel when you think about Republicans?

		**1 – not at all**	**2**	**3**	**4**	**5**	**6**	**7- very strongly**
**1.**	Angry							
**2.**	Afraid							
**3.**	Hopeful							
**4.**	Enthusiastic							
**5.**	Not relevant to me							

32) Are you planning to vote, or have you already voted, in the 2020 presidential election?
- Yes
- No
- Don't know

33) [TO THOSE WHO ANSWERED 'YES' or DK in q21]: Which method are you choosing this year to cast your ballot?

- Absentee voting or voting by mail
- Early voting
- In-person voting on November 3rd
- Don’t know

34) [TO THOSE WHO ANSWERED ’YES’ or DK in q21]: For whom do you plan to (or have) vote(d) for in this 2020 presidential race?

- Donald Trump
- Joe Biden
- Another candidate
- Don’t plan to vote
- Don’t know
